# Psychological and sexological characteristics of rape perpetrators - first report

**DOI:** 10.1192/j.eurpsy.2023.1875

**Published:** 2023-07-19

**Authors:** R. M. Kowalczyk, A. Daren, A. Hejmej, M. Lesniak, K. Waszynska

**Affiliations:** 1 Psychology, Andrzej Frycz Modrzewski Krakow University, Krakow; 2 Psychology, Adam Mickiewicz University, Poznan, Poland

## Abstract

**Introduction:**

For many years, the crime of rape has been the focus of attention of numerous representatives of criminal law doctrines, psychologists and sexologists, and is also part of the considerations of courts and penitentiary centers.

Statistical reports made available by the police clearly indicate that in recent years the problem of sexual crime has been growing, especially crimes consisting in committing a forbidden act such as rape or sexual murder.However, complete and objective data on the prevalence of sexual offenses are very difficult to obtain. Data from the judiciary statistics reveal only a narrow part of the problem of rape.

**Objectives:**

The project involves the analysis of the collected research results, the result of which will be the preparation of a report - a psychological and sexological profile - the characteristics of the convicts. The indicated analysis will be the result of research conducted in several penal facilities in the territory of Poland, including: Kraków, Warsaw, Poznań and others. on a research group comprising about 15% of the total number of prisoners for this crime.

**Methods:**

The source of data on sexual offenders will be data obtained as a result of direct examinations of persons charged with rape, forensic psychiatric opinions, forensic psychological opinions and sexological opinions issued in their cases based on psychiatric and sexological tests commissioned by the court. The research material comes from the files of the perpetrators made available for the purposes of the study by the Central Board of the Prison Service. The authors of the project created a comprehensive perpetrator questionnaire containing a range of data including items on life line, origin, education, work, sexual preferences, sexual life, sexual initiation, psychiatric diagnoses and disorders of sexual preferences and personality.

**Results:**

A preliminary review of the available material gives grounds to conclude that the data it contains is comprehensive and has enormous scientific value. The created tool (based on a comprehensive analysis of the material) by the research team also includes items on the course of the act itself, the method of searching for victims, the method of persuading or forcing victims to submit to sexual activities, the repetition of the act and, finally, the perpetrator’s relationship to the criminal act committed by him.

The final report will be presented at the EPA conference.

**Conclusions:**

The presented report will allow to get to know the characteristic elements of the psychological and sexological profile is a necessary condition for developing an effective strategy of social rehabilitation interactions.

**Disclosure of Interest:**

None Declared

